# The relationship between adipose tissue RAAS activity and the risk factors of prediabetes in different ethnicities: A protocol for a systematic review and meta-analysis

**DOI:** 10.1097/MD.0000000000033095

**Published:** 2024-05-24

**Authors:** Bongeka Cassandra Mkhize, Palesa Mosili, Nomusa Mzimela, Phikelelani Sethu Ngubane, Andile Khathi

**Affiliations:** a University of KwaZulu-Natal, Westville, KwaZulu, Natal, South Africa.

**Keywords:** adiposity, body mass index (BMI), lipotoxicity, meta-analysis, obesity, prediabetes, renin angiotensin aldosterone system, Systematic review

## Abstract

**Background:**

The incidence and prevalence of prediabetes has become a global concern. The risk factors of prediabetes, such as insulin resistance, adiposity, lipotoxicity and obesity, in conjunction with the alteration of the renin-angiotensin-aldosterone system (RAAS), have been positively correlated with the high morbidity and mortality rate. Thus, this systematic review seeks to establish the relationship between the risk factors of prediabetes, namely insulin resistance adiposity, lipotoxicity, obesity and the RAAS. Therefore, a synthesis of these risk factors, their clinical indicators and the RAAS components will be compiled in order to establish the association between the RAAS alteration and obesity in prediabetic patients.

**Methods:**

This protocol for a systematic review was developed in compliance with the Preferred Reporting Items for Systematic Review and Meta-Analysis Protocols (PRISMA-P) standards. This will be accomplished by searching clinical Medical Subject Headings categories in MEDLINE with full texts, EMBASE, Web of Science, PubMed, Cochrane Library, Academic Search Complete, ICTRP and ClinicalTrial.gov. Reviewers will examine all of the findings and select the studies that meet the qualifying criteria. To check for bias, the Downs and Black Checklist will be used, followed by a Review Manager v5. A Forrest plot will be used for the meta-analysis and sensitivity analysis. Furthermore, the strength of the evidence will be assessed utilizing the Grading of Recommendations Assessment, Development, and Evaluation procedure (GRADE). The protocol has been registered with PROSPERO CRD42022320252. This systematic review and meta-analysis will include published randomized clinical trials, observational studies and case-control studies from the years 2000 to 2022.

## 1. Introduction

Prediabetes is a chronic asymptomatic metabolic disorder characterized by impaired fasting glucose and impaired glucose tolerance (IGT).^[1]^ Prediabetes has become a global health burden as the prevalence and incidence of IGT were projected to be 7.3% of the adult population in 2017 and expected to increase to 8.3% of the global adult population, equivalent to 587 million people by the year 2045.^[2]^ The International Diabetes Federation estimated an increase in the prevalence of diabetes in Africa from 14.2 million recorded in 2015 to 34.2 million in 2040.^[3]^ This escalation is concerning as prediabetes often precedes the onset of type 2 diabetes (T2D).^[4]^ T2D is regarded as one of the most significant contributors to death worldwide; 1.6 million deaths were estimated to be caused by T2D in 2016. In the year 2000, 5.5% of South Africans had diabetes and 4.3% of deaths, an equivalent of 22 412 deaths, were attributable to diabetes, making it the 5^th^ highest cause of death in the country.^[3]^ The high mortality rate noted in patients with T2D is directly proportional to the incidence of various comorbidities associated with modulation of the renin-angiotensin-aldosterone system (RAAS), such as insulin resistance and obesity.^[5,6]^ The prevalence of obesity surged from 19.7% to 23.6% between 2008 and 2017, with a significant increment in prevalence for those aged 19 to 24 years. Between the ages of 19 and 50, the percentages of upward transitions to overweight or obese categories grew exponentially.^[7]^ The development of obesity and overweight are preceded by an increase in body mass index (BMI).^[8]^ In 2008, the average BMI at the population level was estimated at 26.9 kg/m^2^ among males (vs a world average of 28.8 kg/m^2^) and 29.5 kg/m^2^ among females (vs a world average of 24.1 kg/m^2^) in South Africa.^[9]^ The increase in BMI in South Africa was reported to be due to the adoption of high-calorie diets and sedentary lifestyles, which are the main contributors to the development and progression of prediabetes.^[7,10]^

As a result of the positive correlation between the incidence of pre-diabetes and the comorbidities noted in prediabetes and T2D, USD 3.4 billion was spent on diabetes in Africa in 2015 and was estimated to rise to USD 3.5 billion in 2040^[11]^ Several studies have reported insulin resistance, hypercholesterolemia, lipotoxicity and obesity in patients in a pre-diabetic state.^[12]^ However, the impact of the risk factors noted in pre-diabetes, namely insulin resistance and obesity associated with the modulation of RAAS, have not been comprehensively and systematically evaluated. Therefore, this study aims to explore the burden of the risk factors of prediabetes in relation to the alteration of RAAS in prediabetic patients by systematically reviewing the risk markers/ clinical indicators for these risk factors in pre-diabetics.

## 2. Study objectives

• To determine local RAAS activity in prediabetic patients by comparison to healthy controls.
• To evaluate the relationship between RAAS components and the risk factors for prediabetes such as insulin resistance, lipotoxicity adiposity and obesity.
• In order to satisfy the above objectives, the risk markers for the mentioned risk factors will be assessed in addition to the activity of RAAS in adipose tissue and in prediabetes patients by comparison to the non-prediabetic.

## 3. Methods

The Preferred Reporting Items for Systematic Review and Meta-Analysis Protocols (PRISMA-P) criteria were used in the preparation of this systematic review protocol.^[13]^

### 3.1. Systematic review registration

The protocol has been registered with PROSPERO with registration number “CRD42022320252”

### 3.2. Eligibility criteria

In 2003, the American Diabetes Association revised its diagnosis criteria.^[14,15]^ The American Diabetes Association recommended reducing the Impaired Fasting Glucose threshold from 6.1 mmol/l (110 mg/dL) to 5.6 mmol/l (100mg/dL) while maintaining the diagnostic criteria for diabetes and IGT.^[14,15]^ The World Health Organization and the International Diabetes Federation decided to amend their current recommendations for the classification and diagnosis of diabetes and intermediate hyperglycemia.^[15]^ Henceforth, randomized controlled clinical trials, observational studies and case-control studies from the year 2000 to 2022 published in English will be included in this review. Only articles published in peer-reviewed journals with more than 100 participants will be accepted in this review as they are more reliable.

### 3.3. Participants

In this systematic review, randomized controlled trials, observational studies, and case-control studies reporting on prediabetic individuals. The participants will be both males and females from all ethnic groups who are 18 years and older. Furthermore, participants are required to satisfy at least one of the following diagnoses, fasting blood glucose: 5.6 to 7.0 mmol/L; 2 h postprandial blood glucose: 7.8 to 11.0 mmol/L with glycated hemoglobin: 5.7% to 6.4% in order to be considered for this systematic review

### 3.4. Exposure

Randomized control trials, observational studies, and case-control studies that report on the risk factors of prediabetes, namely, obesity, adiposity, lipotoxicity and insulin resistance in association to changes in the RAAS components in a prediabetic state, will be eligible for the systematic review. Studies that inform on the clinical markers of any of the mentioned risk factors (obesity, adiposity, lipotoxicity and insulin resistance) in a prediabetic state and its relation to alteration of the RAAS components will be considered for this review.

### 3.5. Comparators

Prediabetic patients defined by IGT, impaired fasting glucose and glycated hemoglobin and non-prediabetic patients.

### 3.6. Outcomes

1. Primary Outcomes
  - Activity of the RAAS components viz; renin, angiotensin-converting enzyme, angiotensin II type 1 receptor, angiotensin II, angiotensin 1-7, angiotensin-converting enzyme 2 and aldosterone determined by the protein concentration and angiotensin II type 1 receptor expression.
  - Comorbidities regarded as risk factors for prediabetes and type 2 diabetes, namely, insulin resistance, lipotoxicity, adiposity and obesity (reported as OR).
2. Surrogate outcomes
  - Obesity risk markers such as adipokines/ adipocytokines expression/ triglycerides/ cholesterol concentration.

### 3.7. Search strategy

Electronic search strategies with the assistance of a subject librarian will be implored using Medical Subject Headings with the keywords such as the renin angiotensin aldosterone system OR angiotensin II AND “Prediabetes” or “impaired glucose tolerance” or “impaired fasting glucose” AND “adipose” or “adiposity” or “body mass index” or “bmi” or “obese” or “obesity” or “overweight” or “weight.” Databases such as MEDLINE, EMBASE, Web of Science, Cochrane Library, Academic Search Complete, ICTRP and ClinicalTrial.gov will be utilized to identify published studies highlighting the relationship between prediabetes and the mentioned comorbidities.

### 3.8. Study selection process

A broad search strategy will be implemented to ensure that all the relevant studies are included in the review. Based on the relevance of the title and abstract article will be screened and excluded. Furthermore, only full-text articles that comply with the inclusion criteria will be selected. The level of inter-rater agreement will be assessed using Cohen kappa inter-rater reliability.^[16]^

The articles and abstracts from the search will be evaluated for relevance and categorized into 1 of 3 groups: not pertinent, pertinent, or possibly pertinent. The pertinent full text will be thoroughly assessed in order to identify the studies applicable to this review. Endnote X20 reference manager database will be used to exclude duplicates.

### 3.9. Data collection process

BCM and PM will independently screen the titles, abstracts and the selected full-text articles against the eligibility criteria to extract data. Discrepancies between the researchers will be analyzed and resolved to reach a consensus. If a consensus cannot be achieved, an arbitrator will be consulted. Furthermore, an excel spreadsheet with the study characteristics will be included in order to manage the data.

### 3.10. Data simplification

The relevant research articles that will be included in the review will be grouped according to the mentioned comorbidity and the associated risk markers (e.g., obesity and adiponectin). Each table will consist of studies that report on the RAAS components, the specific risk factor (e.g., obesity and insulin resistance) and the associated markers.

### 3.11. Risk of bias in individual studies

BCM and PM will independently assess the risk of bias using the 4 domains of the Downs and Black checklist, namely, reporting bias (10 items), external validity (3 items), internal validity (6 items), and selection bias (7 items). The results will be graded as excellent (25–26), good (20–24), moderate (14–19), poor (11–13), and extremely bad (<10).^[17]^ Additionally, BCM, PM and NCM will evaluate the included studies, and independent reviewers (PSN and AK) will arbitrate any disagreements.

### 3.12. Data synthesis

The major outcomes of the study will be summarized in a summary of findings table using the GRADE pro tool. Furthermore, data will be analyzed with a forest plot from Review Manager version 5.4 to do a meta-analysis if the included studies are homogenous with regards to the type of risk markers.^[18,19]^ The statistical heterogeneity in the selected studies will be assessed with RevMan forest plot using the I^2^ and Chi-squared statistical test to test if an association between the condition (prediabetes) and the risk factors exists. An I^2^ of <25% will be considered low heterogeneity, between 25% and 50% as moderate, and >50% as high heterogeneity.^[19]^ The forest plot will also provide an odds ratio and confidence interval to quantify the effective size of the association., with solid lines indicating the 95 % confidence interval. Each of the included studies will be denoted on the y-axis by a horizontal line with the lead investigator and year of the study indicated.

### 3.13. Sensitivity analysis

Studies having an I^2^ > 50 will be assessed for potential sources of heterogeneity using a sensitive analysis and by excluding studies that are judged to be at high risk for bias.^[20]^ Furthermore, a significant overlap between the confidence intervals will suggest substantial homogeneity.

### 3.14. Strength of evidence

To examine the quality of the overarching evidence, the Grading of Recommendations, Assessment, Development, and Evaluation (GRADE) assessment instrument will be utilized.^[21]^ Study limitations, indirectness of outcomes and publishing or reporting bias will all be considered in determining the quality of evidence. Each outcome evidence will be graded as high, moderate, low, or extremely low. BCM, PM and NCM will independently review the quality of the included studies, PSN and AK will arbitrate.

## 4. Discussion and conclusion

This systematic review will provide insight into the relationship between the prevalence of the risk markers and factors of these comorbidities in prediabetes. Furthermore, this review aims to highlight the correlation between such as obesity and insulin resistance in prediabetes and the modulation of the RAAS.

## Acknowledgments

The authors would like to express gratitude to National Research Foundation and the University of KwaZulu-Natal College of Health Science.

## Author contributions

**Conceptualization:** Bongeka Cassandra Mkhize, Andile Khathi.

**Data curation:** Nomusa Mzimela.

**Investigation:** Bongeka Cassandra Mkhize, Palesa Mosili.

**Methodology:** Bongeka Cassandra Mkhize, Nomusa Mzimela.

**Project administration:** Andile Khathi.

**Resources:** Andile Khathi.

**Supervision:** Phikelelani Sethu Ngubane, Andile Khathi.

**Writing – original draft:** Bongeka Cassandra Mkhize, Palesa Mosili.

**Writing – review & editing:** Bongeka Cassandra Mkhize, Phikelelani Sethu Ngubane, Andile Khathi.
